# Supplementary material for: CGRP, adrenomedullin and adrenomedullin 2 display endogenous GPCR agonist bias in primary human cardiovascular cells
Source: Commun Biol. 2021 Jun 23;4:776. doi: 10.1038/s42003-021-02293-w (PMC8222276; doi:10.1038/s42003-021-02293-w)
Supplement: Supplementary file 2 — Supplementary Information [file 42003_2021_2293_MOESM2_ESM.pdf]

Supplementary information for

CGRP, adrenomedullin and adrenomedullin 2 display endogenous GPCR agonist bias in primary human cardiovascular cells

Ashley J. Clark[1], Niamh Mullooly[2], Dewi Safitri[1,3], Matthew Harris[1], Tessa de Vries[4], Antoinette MaassenVanDenBrink[4], David R. Poyner[5], Davide Gianni[2], Mark Wigglesworth[6], Graham Ladds[1]

Address for correspondence:
Dr Graham Ladds, Department of Pharmacology, University of Cambridge, Tennis Court Road, Cambridge, CB2 1PD Tel; +44 (0) 1223 334020. Email: grl30@cam.ac.uk.

Supplementary Figures S1-S7
Supplementary Tables S1-S3

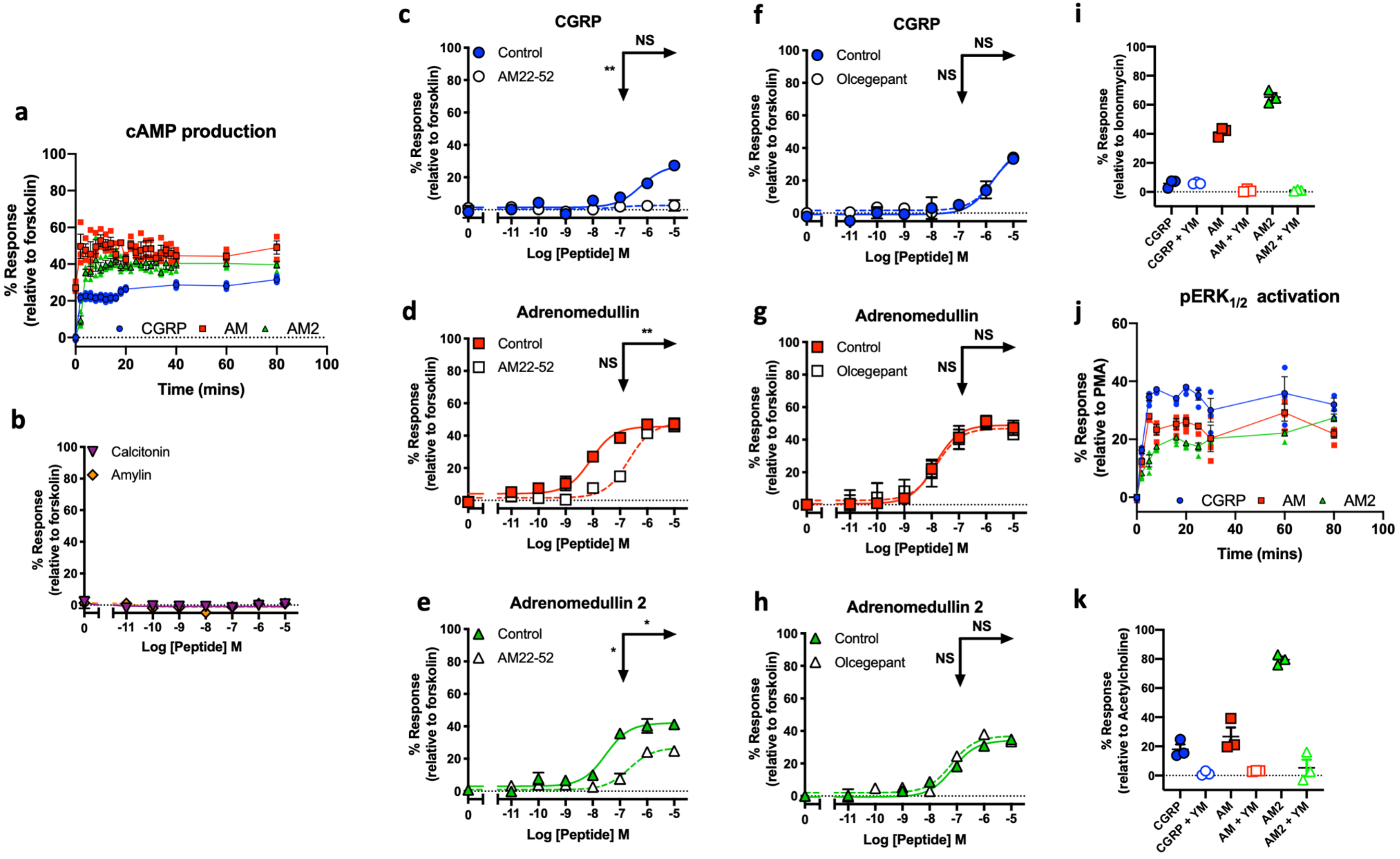

Supplemental Figure 1. Pharmacological assessment of signalling properties in HUVECs.

a) Time-dependent analysis of cAMP accumulation in response to stimulation by 1μM CGRP, AM and AM2 in HUVECs. Responses normalised to 100μM forskolin. **b)** Characterisation of cAMP accumulation in response to stimulation by calcitonin and amylin in HUVECs

relative to forskolin (100μM). **c-f**) Characterisation of cAMP accumulation in response to stimulation by CGRP (**c**,**f**), AM (**d**,**g**) and AM2 (**e**,**h**) in HUVECs in the presence and absence of 100nM AM22-52 (**c-e**) or 100 nM Olcegepant (**f-h**). Data are analysed using a three-parameter non-linear regression curve. Statistical significance determined compared to control using an unpaired Student's t test with Welch's correction (*, $p<0.05$; **, $p<0.01$; ***, $p<0.001$ ****, $p<0.0001$). NS denotes no statistical significance observed. Rows show pEC_{50}, and Vertical arrows show E_{max} statistical significance. **i**). Characterisation of Ca^{2+}_i mobilisation with and without YM-254890 relative to ionomycin (10μM) and plotted as E_{max} values. **j**) Time-dependent analysis of $ERK_{1/2}$ activation in response to stimulation with 1μM CGRP, AM and AM2 in HUVECs. Responses normalised to 10μM PMA. **k**) Characterisation of NO release with and without YM-254890 relative to acetylcholine (10μM) and plotted as E_{max} values ± SEM. **a-h** are mean ± SEM of 5 independent repeats. **i-k** are mean ± SEM of 3 independent replicates.

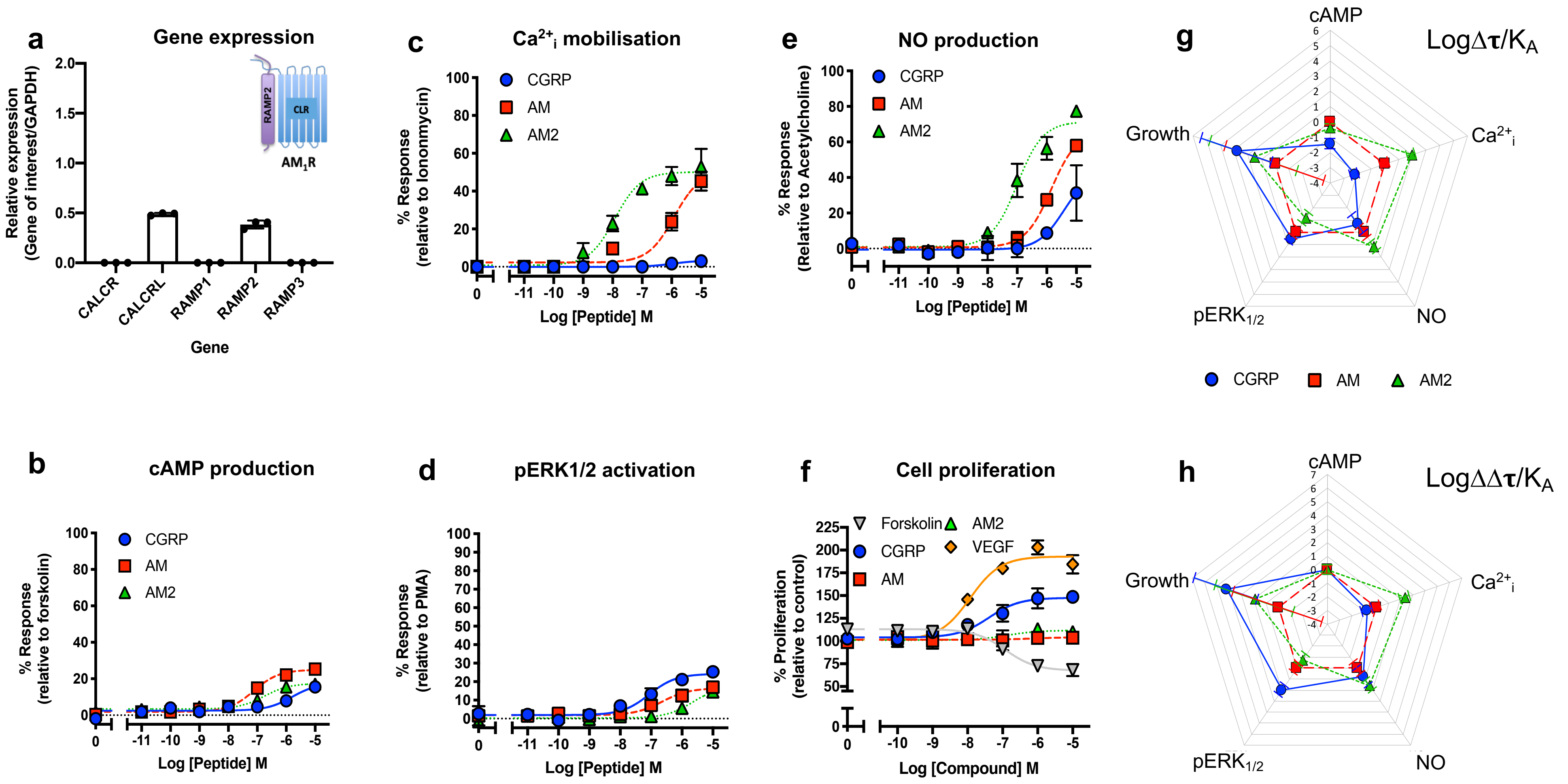

Supplementary Figure 2. CGRP family peptide signalling bias in HUAECs. **a**) Expression of *CALCR*, *CALCRL*, *RAMP1*, *RAMP2*, and *RAMP3* genes in HUAECs. Data represent mean ± SEM of at 3 independent experiments relative to GAPDH expression. **b-g)** Dose-response curves were constructed for HUVECs stimulated with CGRP, AM or AM2 and the cAMP levels quantified relative to forskolin (100μM) (**b**), mobilisation of Ca^{2+}_i relative to ionomycin (10μM) (**c**), intracellular $ERK_{1/2}$ phosphorylation relative to PMA (10μM) (**d**), total NO production relative to acetylcholine (10μM) (**e**), and extent of cell proliferation (after 72h) relative to vector treated control and VEGF (**f**). Data are analysed using a three-parameter non-linear regression curve or the operational model of receptor agonism[27]. **g-h**) Signalling

bias plots were calculated as $\Delta Log(\tau/K_A)$ (**g**) or $\Delta\Delta Log(\tau/K_A)$ (**H**) for each agonist and for each signalling pathway. Determination of values requires normalisation to a reference agonist (AM) alone in **G**, while for **H** values were normalised to both a reference agonist (AM) and a reference pathway (cAMP). All data represent mean ± SEM of 5 independent experiments.

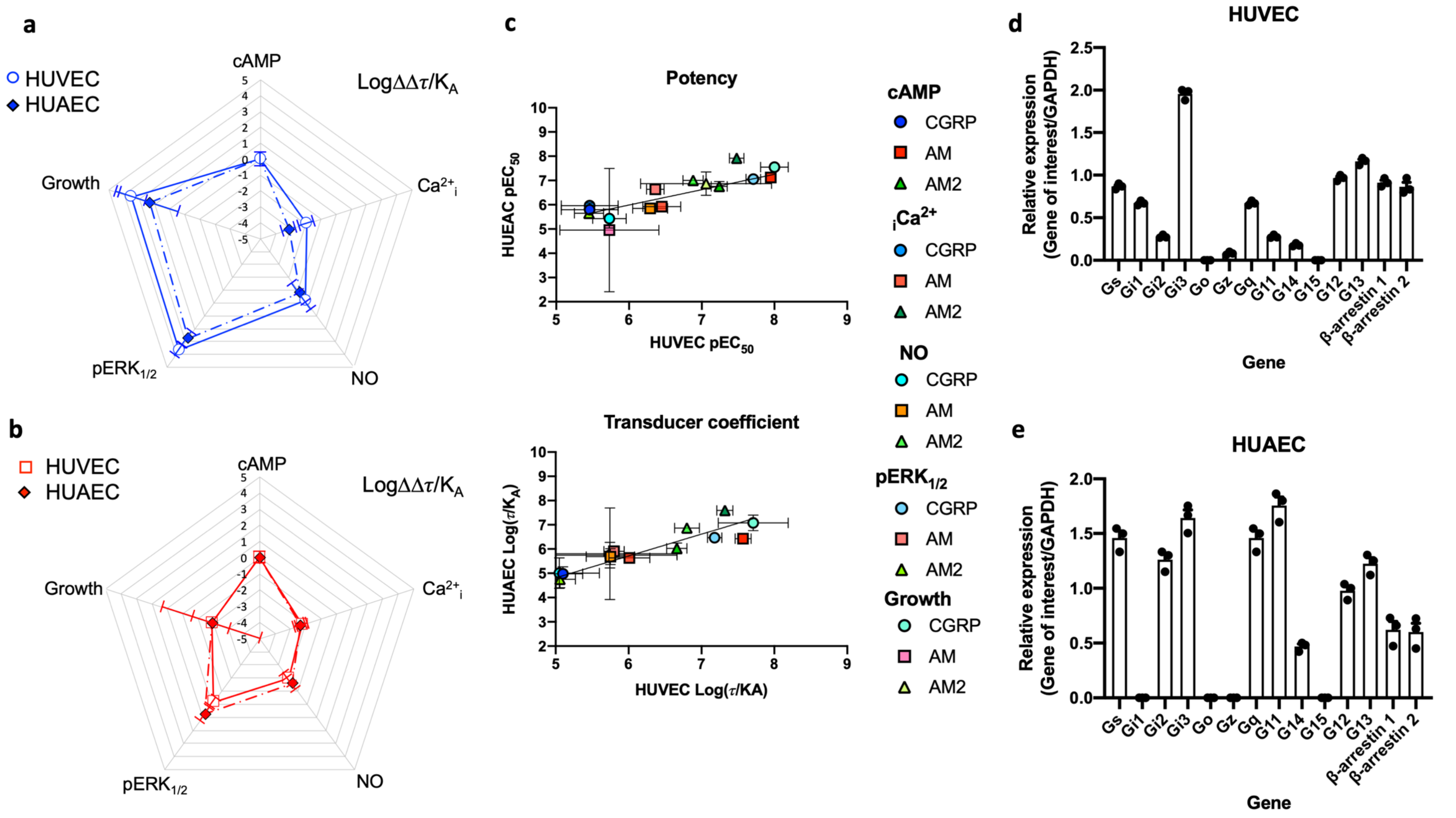

Supplemental Figure 3. Comparison of HUVEC and HUAEC signalling properties and G protein content

a) Signalling bias plots were calculated as ΔΔLog(τ/K_A) for CGRP in the two cell lines, HUVECs and HUAECs for each pathway. Values have been normalised to a reference agonist (AM2) and the reference pathway (cAMP) for both cell lines. **b**) as for **a** except the calculated

values are for AM. **c**) the correlation of log agonist potencies ± SEM and transduction coefficient (τ/K_A) ± SEM for CGRP, AM and AM2 stimulated cAMP accumulation, mobilisation of $Ca^{2+}{}_i$, NO production, intracellular $ERK_{1/2}$ phosphorylation and cell proliferation in HUVECs and HUAECs was analysed by a scatter plot and Pearson's correlation coefficients (*r*) were calculated. Significant positive correlation was observed for both potency and transduction coefficient between HUVECs and HUAECs as indicated by the presence of the line. **d-e)** Expression of GPCR accessory protein genes in HUVECs (**d**) and HUAECs (**e**). Data represent mean ± SEM of 3 independent experiments relative to GAPDH expression.

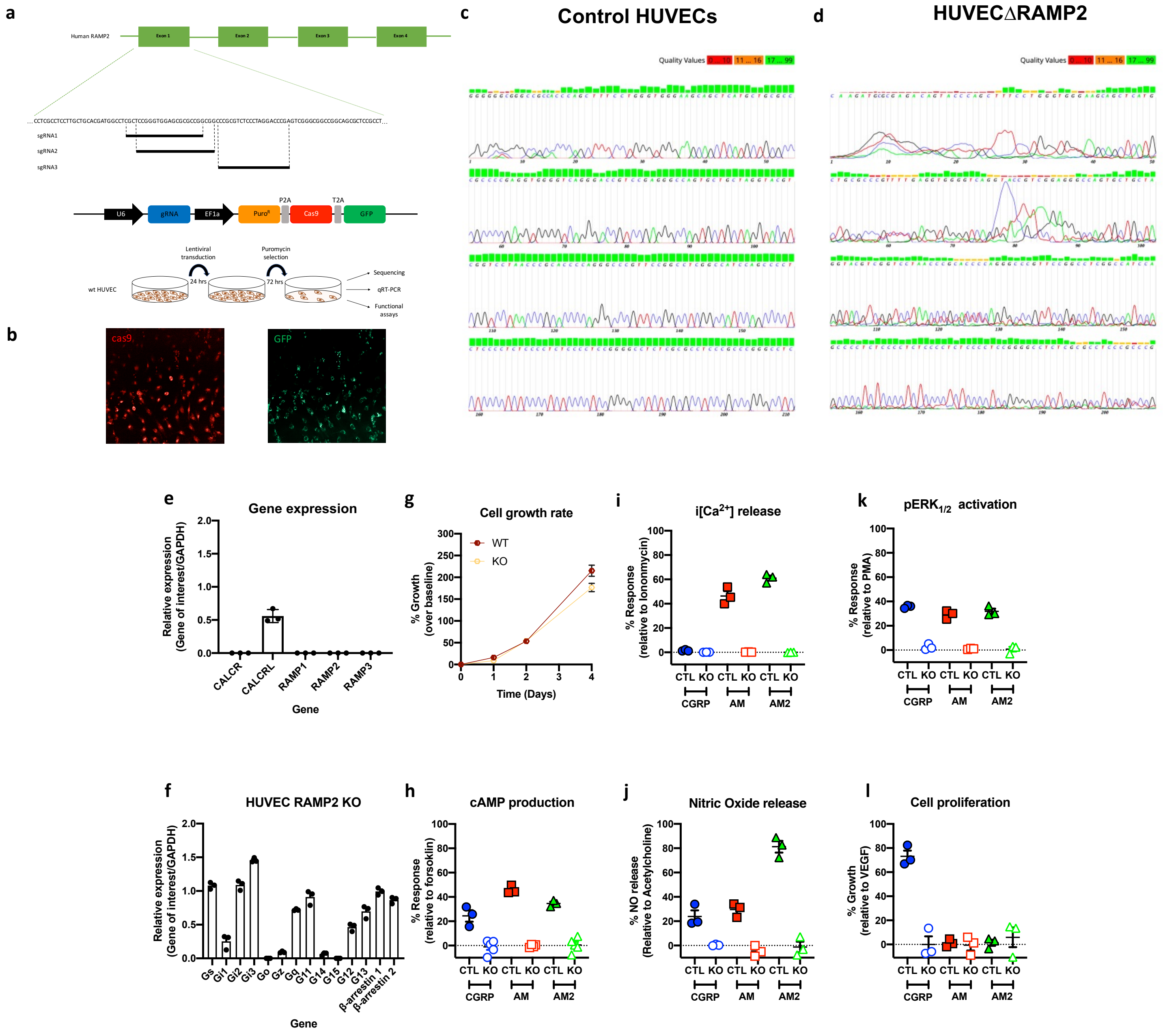

a
Human RAMP2
Exon 1
Exon 2
Exon 3
Exon 4
sgRNA1
sgRNA2
sgRNA3
U6
gRNA
EF1a
Puro
P2A
Cas9
T2A
GFP
Lentiviral transduction
Puromycin selection
24 hrs
72 hrs
wt HUVEC
Sequencing
qRT-PCR
Functional assays
b
cas9
GFP
c
Control HUVECs
Quality Values
d
HUVECΔRAMP2
Quality Values
e
Gene expression
Relative expression (Gene of interest/GAPDH)
CALCR
CALCRL
RAMP1
RAMP2
RAMP3
Gene
g
Cell growth rate
% Growth (over baseline)
WT
KO
Time (Days)
i
i[Ca2+] release
% Response (relative to Ionomycin)
CTL
KO
CGRP
AM
AM2
k
pERK1/2 activation
% Response (relative to PMA)
f
HUVEC RAMP2 KO
Relative expression (Gene of interest/GAPDH)
Gs
Gi1
Gi2
Gi3
Go
Gz
Gq
G11
G14
G15
G12
G13
β-arrestin 1
β-arrestin 2
Gene
h
cAMP production
% Response (relative to forsokilin)
j
Nitric Oxide release
% NO release (Relative to Acetylcholine)
l
Cell proliferation
% Growth (relative to VEGF)

Supplemental Figure 4. Generation and confirmation of RAMP2 knockout HUVEC cell line by CRISPR-Cas9 gene editing and signalling characteristics and G protein content of HUVEC∆RAMP2 cell line. a) Schematic of the three gRNA sequences targeting the 1st exon of the WT RAMP2 gene and the lentiviral vector expressing gRNA, Puromycin resistance, GFP and Cas9 combined with the workflow for generation of gene edited HUVEC cell pool. **b)** Confocal imaging of HUVEC cells following transduction of lentivirus and puromycin selection. Showing high levels of Cas9 expression and nuclear localization and high levels of GFP expression with cytoplasmic expression. **c-d**) Genomic Sequencing results provided by Euorgentics for section of exon 1 of RAMP2 gene containing sgRNA target sites for Control HUVECs © vs same section in CRISPR-Cas9 RAMP2 knockout HUVECs (**d)** sequenced using the RAMP2 primer: GCCTATTTTCGGAGGGTCTC. The sequencing results from the in CRISPR-Cas9 RAMP2 knockout HUVECs demonstrates heterogeneity indicative of a mixed pool of cells with different genome edits to the RAMP2 open reading frame. **e)** Expression of *CALCR, CALCRL, RAMP1, RAMP2,* and *RAMP3* genes in RAMP2 knockout HUVECs. **f**) Expression of GPCR accessory protein genes in KO HUVECs. For **e-f**, data represent mean ± SEM of 3 independent experiments relative to GAPDH expression. **g)** Characterisation of cell growth rate in WT and RAMP2 KO HUVECs over 4 days relative to day zero. **h-l**) WT and RAMP1 null HUVECs were stimulated with CGRP, AM and AM2 and the maximal response (E_{max}) quantified for **h**) cAMP accumulation relative to 100μM forskolin, **i**) $Ca^{2+}{}_i$ mobilisation relative to 10μM ionomycin, **j**) total NO production relative to 10μM acetylcholine, **k**) intracellular $ERK_{1/2}$ phosphorylation relative to 10μM PMA, **l**) cell proliferation relative to 10μM VEGF. All pathways measured in response to stimulation by CGRP, adrenomedullin (AM) and adrenomedullin 2 (AM2). All data represent E_{max} ± SEM of 3 independent experiments.

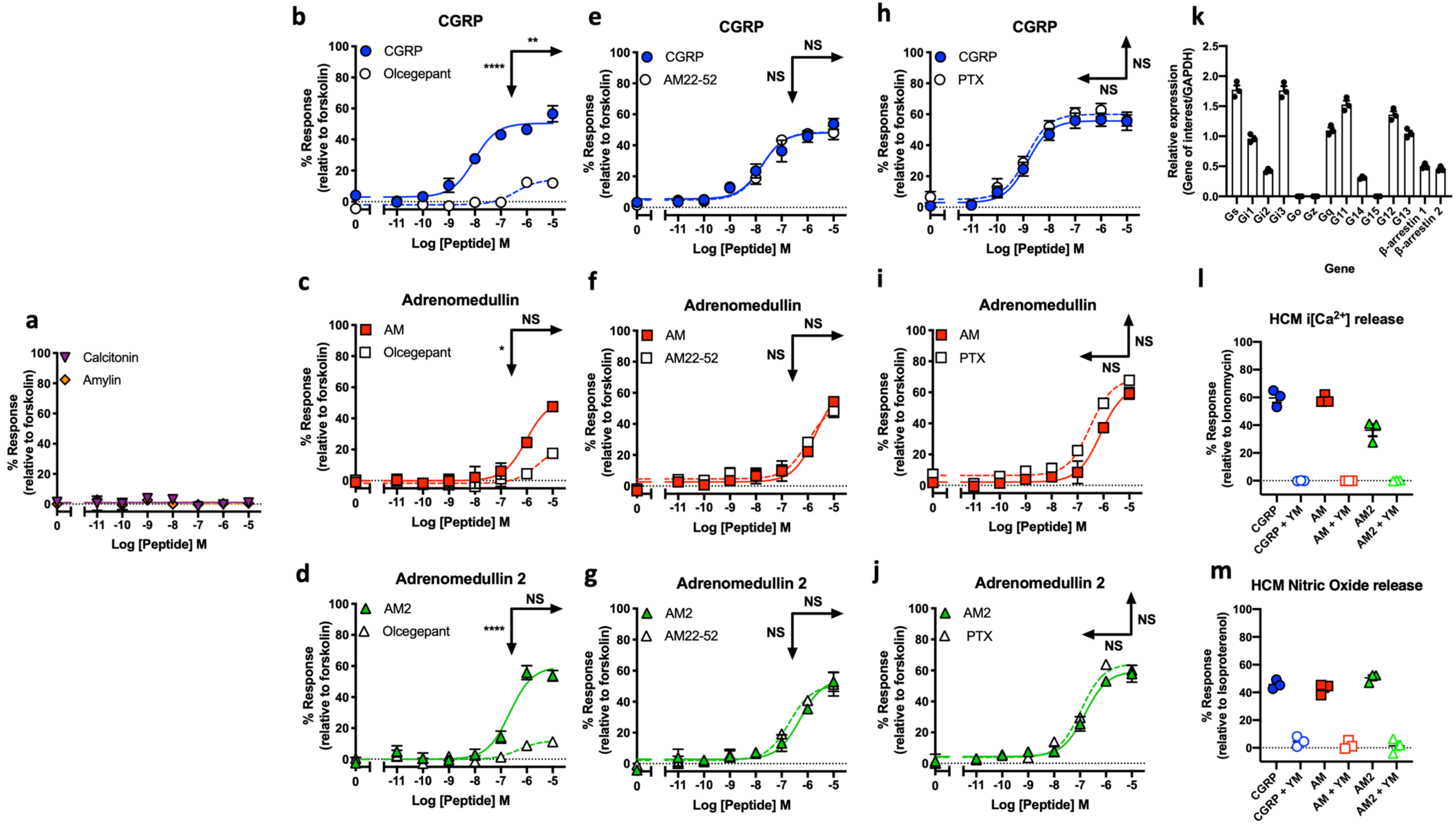

Supplemental Figure 5. Pharmacological assessment of signalling properties in HCMs.

a) Characterisation of cAMP accumulation in response to stimulation by calcitonin and amylin in HCMs relative to forskolin (100 μM). **b-g**) Characterisation of cAMP accumulation in response to stimulation by CGRP (**b,e**), AM (**c,f**) and AM2 (**d,g**) in HCMs in the presence and absence of 100nM Olcegepant (**b-d**) or AM22-52 (**e-g**). All relative to 100μM forskolin. **h-j**) Characterisation of cAMP accumulation in

response to stimulation by CGRP (**h**), AM (**i**) and AM2 (**j**) with and without PTX treatment relative to forskolin (100μM) Data are analysed using a three-parameter non-linear regression curve. Statistical significance determined compared to control using an unpaired Student's t test with Welch's correction (*, $p<0.05$; **, $p<0.01$; ***, $p<0.001$ ****, $p<0.0001$). NS denotes no statistical significance observed. All values are calculated from 3 individual data sets. Horizontal arrows show pEC_{50}, and vertical arrows show E_{max} statistical significance. **k**) Expression of GPCR accessory protein genes in HCMs. Data represent mean ± SEM from four independent experiments relative to GAPDH expression. **l**) Characterisation of $Ca^{2+}{}_i$ mobilisation with and without YM-254890 relative to Ionomycin (10μM) and plotted as E_{max} values. **m)** Characterisation of NO release with and without YM-254890 relative to acetylcholine (10μM) and plotted as E_{max} values. **a-j** data represent mean ± SEM of 4 independent experiments. Data in **l-m** represent E_{max} ± SEM of 3 independent experiments.

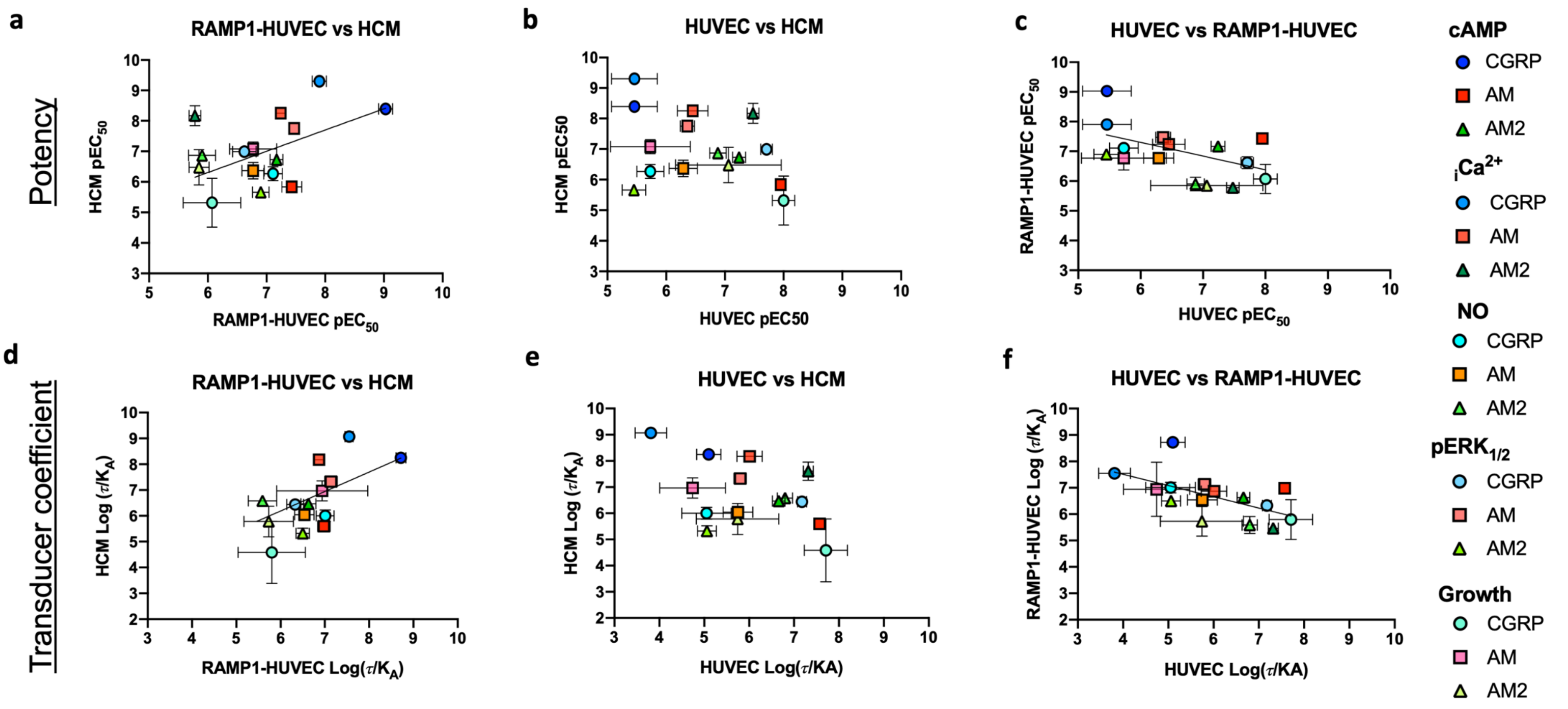

Supplemental Figure 6. Correlation of signalling properties between RAMP1-HUVEC, HCMs and HUVECs.
a-c) The correlation of Log agonist potencies ± SEM for CGRP, AM and AM2 stimulated cAMP accumulation, mobilisation of $Ca^{2+}{}_i$, NO production, intracellular $ERK_{1/2}$ phosphorylation and cell proliferation in RAMP1 expressing HUVECs and HCMs (**a**), HUVECs and HCMs (**b**) and HUVECs and RAMP1 expressing HUVECs (**c**) was analysed by a scatter plot and Pearson's correlation coefficients (*r*) were calculated. A significant positive correlation was observed for RAMP1 expressing HUVECs and HCMs. The presence of a line indicates a positive correlation. **d-f**) as for **a-c** except the transduction coefficient Log (τ/K_A) was calculated. A significant positive correlation was observed for RAMP1 expressing HUVECs and HCMs as indicated by the presence of a line.

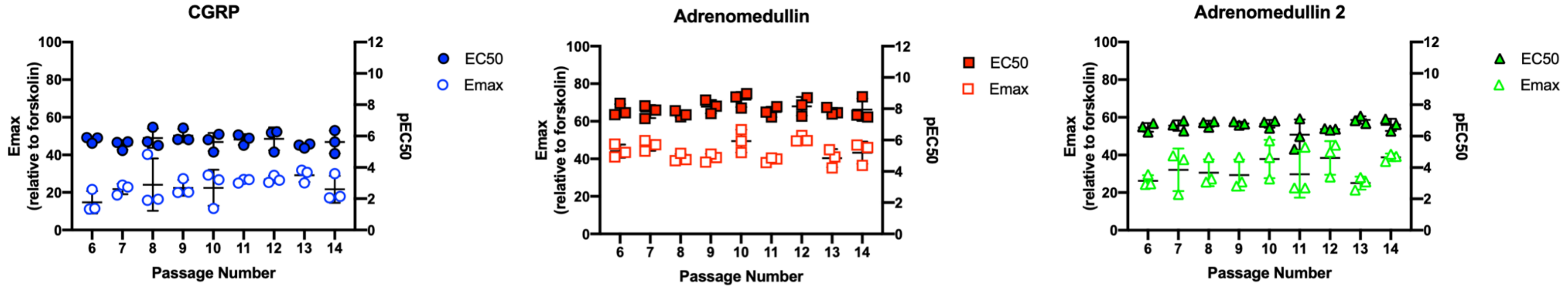

Supplemental Figure 7. Quantification of CGRP signalling in HUVECs is unaffected by passage number Characterisation of cAMP accumulation in response to stimulation by CGRP, AM and AM2 in HUVECs relative to 100μM forskolin across multiple passages (passage 6 - passage 14). Data are analysed using a three-parameter non-linear regression curve, and individual pEC_{50}/E_{max} values and mean ± SEM are plotted for 3 independent experiments.

Supplemental Table 1 Potency (pEC_{50}), effector Maximum (E_{max}), affinity (pK_A) and coupling efficacy (log τ) values for all signalling pathways various agonists measured in HUVECs and HUAECs.

	HUVEC			HUAEC		
	**cAMP**					
	**CGRP**	**AM**	**AM2**	**CGRP**	**AM**	**AM2**
**pEC_{50}[a]**	5.46±0.39****	7.95±0.07	7.24±0.11	5.80±0.27***	7.12±0.16	6.75±0.20
**E_{max}[b]**	27.14±6.75	45.17±1.17	32.75±1.21	17.51±2.46*	24.97±1.51	17.46±1.21*
**pK_A[c]**	5.61±0.25****	7.67±0.09	6.99±0.14****	5.72±0.28***	6.95±0.14	6.78±0.21
**logτ[d]**	-0.51±0.09***	-0.10±0.07	-0.33±0.03	-0.74±0.09*	-0.52±0.03	-0.75±0.05*
	**Ca^{2+}_i**					
**pEC_{50}[a]**	5.45±0.33**	6.47±0.26	7.48±0.10*	5.96±0.14	5.92±0.17	7.92±0.14****
**E_{max}[b]**	2.26±0.54****	34.72±3.71	66.37±2.66****	3.36±0.29****	50.35±4.95	50.11±3.22
**pK_A[c]**	5.44±0.34	6.29±0.27	7.03±0.10	4.74±0.14	5.65±0.21	7.61±0.13****
**logτ[d]**	-1.63±0.10****	-0.28±0.07	0.29±0.05****	-1.29±0.04****	-0.01±0.08	-0.01±0.05
	**pERK1/2**					
**pEC_{50}[a]**	7.71±0.10***	6.36±0.12	5.46±0.2**	7.06±0.18	6.64±0.15	5.65±0.21*
**E_{max}[b]**	35.17±1.13	30.16±1.75	33.30±4.59	24.43±1.62*	16.48±0.94	17.59±2.44
**pK_A[c]**	7.46±0.10***	6.18±0.13	5.41±0.19**	6.99±0.13	6.66±0.21	5.43±0.33*
**logτ[d]**	-0.28±0.02	-0.38±0.04	-0.35±0.08	-0.52±0.03	-0.75±0.05	-0.67±0.12
	**NO**					
**pEC_{50}[a]**	5.73±0.23	6.29±0.24	6.88±0.14	5.43±0.39	5.85±0.09	7.00±0.18*
**E_{max}[b]**	21.13±2.89	31.92±4.25	82.1±4.62***	43.25±13.02	66.07±2.63	71.48±4.88
**pK_A[c]**	5.64±0.52	6.08±0.31	6.15±0.15	5.10±0.56	5.41±0.30	6.47±0.14
**logτ[d]**	-0.59±0.18	-0.33±0.11	0.65±0.09**	-0.09±0.28	0.28±0.13	0.4±0.07
	**Cell proliferation**					
**pEC_{50}[a]**	8.00±0.19*	5.73±0.68	7.06±0.9	7.55±0.19**	4.96±2.54	6.87±0.48*
**E_{max}[b]**	140.39±2.13****	106.90±3.47	103.74±1.43	147.55±3.07**	119.22±4.44	111.68±1.62
**pK_A[c]**	7.69±0.19	5.69±0.70	7.03±0.90	7.43±0.31	5.30±1.80	6.91±1.87
**logτ[d]**	0.02±0.06**	-0.95±0.22	-1.29±0.20	-0.35±0.07	-1.00±1.79	-1.10±0.26

Data are the mean ± SEM of 3-6 individual data sets.
[a] The negative logarithm of the agonist concentration required to produce a half-maximal response.
[b] The maximal response to the agonist expressed as a percentage of the system maximal as define in the methods.
[c] The negative logarithm of the equilibrium disassociation constant for each ligand generated through use of the operational model of agonism[27].
[d] τ is the coupling efficiency parameter of each ligand.
Statistical significance compared to the cognate ligand AM (*, $p < 0.05$, **, $p < 0.01$, ***, $p < 0.001$, ****, $p < 0.0001$) for each pathway was determined by one-way ANOVA with Dunnett's post-test.

Supplemental Table 2. Potency (pEC_{50}), effector Maximum (E_{max}), affinity (pK_A) and coupling efficacy (log τ) values for all signalling pathways various agonists measured in RAMP1-HUVECs and HCMs.

	RAMP1-HUVEC			HCM		
	**cAMP**					
	**CGRP**	**AM**	**AM2**	**CGRP**	**AM**	**AM2**
**pEC_{50}[a]**	9.03±0.12	7.43±0.17****	7.17±0.11****	8.39±0.12	5.84±0.08****	6.73±0.10****
**E_{max}[b]**	43.2±1.29	38.95±2.08	32.67±1.57**	62.91±1.85	63.05±3.14	61.04±2.5
**pK_A[c]**	8.87±0.11	7.20±0.14****	6.97±0.16****	8.05±0.09	5.38±0.19****	6.29±0.14****
**logτ[d]**	-0.15±0.02	-0.22±0.03	-0.34±0.05**	0.20±0.03	0.22±0.10	0.18±0.06
	**Ca^{2+}_i**					
**pEC_{50}[a]**	7.90±0.12	7.24±0.09***	5.78±0.10****	9.30±0.10	8.25±0.17*	8.17±0.33*
**E_{max}[b]**	51.30±2.06	44.13±1.42*	40.96±2.26**	64.44±1.73	58.12±0.15	36.49±2.31***
**pK_A[c]**	7.52±0.09	6.97±0.10*	5.64±0.18****	8.85±0.15	8.10±0.15	7.91±0.35
**logτ[d]**	0.03±0.03	-0.10±0.03	-0.18±0.06**	0.22±0.05	0.08±0.04	-0.30±0.05***
	**pERK1/2**					
**pEC_{50}[a]**	6.62±0.20	7.47±0.08*	6.90±0.14	6.99±0.11	7.75±0.14*	5.66±0.17**
**E_{max}[b]**	38.69±3.4	47.57±1.48	45.35±2.89	33.02±1.52	40.13±2.02	40.41±4.17
**pK_A[c]**	6.52±0.17	7.16±0.13*	6.56±0.14	6.77±0.15	7.52±0.12*	5.52±0.18**
**logτ[d]**	-0.19±0.05	-0.02±0.04	-0.06±0.05	-0.33±0.04	-0.19±0.03	-0.20±0.08
	**NO**					
**pEC_{50}[a]**	7.11±0.16	6.77±0.19	5.90±0.23**	6.27±0.23	6.37±0.27	6.87±0.11
**E_{max}[b]**	75.15±4.51	56.30±4.5	53.96±7.60	49.97±5.56	46.98±5.85	50.59±2.18
**pK_A[c]**	6.54±0.18	6.43±0.21	5.51±0.29*	5.99±0.22	6.07±0.22	6.55±0.18
**logτ[d]**	0.47±0.09	0.11±0.08	0.08±0.14	0.01±0.08	-0.03±0.08	0.03±0.06
	**Cell proliferation**					
**pEC_{50}[a]**	6.07±0.49	6.77±0.40	5.85±0.17	5.32±0.80	7.08±0.23*	6.48±0.58
**E_{max}[b]**	143.61±12.20	195.27±14.23*	201.29±11.45*	108.8±24.20	160.32±6.82*	114.68±3.58
**pK_A[c]**	5.89±0.70	5.84±0.81	5.02±0.44	5.23±0.90	6.45±0.33	6.38±0.59
**logτ[d]**	-0.09±0.30	1.10±0.64	0.71±0.34	-0.65±0.80	0.52±0.20	-0.60±0.12

Data are the mean ± SEM of 3-6 individual data sets.
[a] The negative logarithm of the agonist concentration required to produce a half-maximal response.
[b] The maximal response to the agonist expressed as a percentage of the system maximal as define in the methods.
[c] The negative logarithm of the equilibrium disassociation constant for each ligand generated through use of the operational model of agonism[27].
[d] τ is the coupling efficiency parameter of each ligand.
Statistical significance compared to the cognate ligand CGRP (*, $p < 0.05$, **, $p < 0.01$, ***, $p < 0.001$, ****, $p < 0.0001$) for each pathway was determined by one-way ANOVA with Dunnett's post-test.

Supplemental Table 3. Transducer coefficients Log(τ/K_A) as determined for all signalling pathways various agonists as measured in HUVECs, HUAECs, RAMP1-HUVEC and HCMs. Values calculated from the data shown in Table S1 and Table S2,

	CGRP	AM	AM2
**HUVEC**			
**cAMP**	5.10±0.27	7.57±0.11	6.66±0.14
**Ca^{2+}_i**	3.81±0.35	6.01±0.28	7.32±0.11
**$pERK_{1/2}$**	7.18±0.10	5.80±0.14	5.06±0.21
**NO**	5.05±0.55	5.75±0.33	6.80±0.17
**Growth**	7.71±0.48	4.74±0.73	5.74±0.92
**HUAEC**			
**cAMP**	4.98±0.29	6.43±0.14	6.03±0.22
**Ca^{2+}_i**	3.45±0.15	5.64±0.22	7.60±0.14
**$pERK_{1/2}$**	6.47±0.13	5.91±0.22	4.76±0.35
**NO**	5.01±0.63	5.69±0.33	6.87±0.16
**Growth**	7.08±0.32	4.30±2.54	5.81±1.89
**RAMP1-HUVEC**			
**cAMP**	8.72±0.11	6.98±0.14	6.63±0.17
**Ca^{2+}_i**	7.55±0.09	6.87±0.10	5.46±0.19
**$pERK_{1/2}$**	6.33±0.19	7.14±0.14	6.50±0.15
**NO**	7.01±0.20	6.54±0.22	5.59±0.32
**Growth**	5.80±0.76	6.94±1.03	5.73±0.56
**HCM**			
**cAMP**	8.25±0.09	5.60±0.21	6.47±0.15
**Ca^{2+}_i**	9.07±0.16	8.18±0.16	7.61±0.35
**$pERK_{1/2}$**	6.44±0.16	7.33±0.12	5.32±0.20
**NO**	6.00±0.23	6.04±0.23	6.58±0.19
**Growth**	4.58±1.20	6.97±0.39	5.78±0.60
