# Supplementary material for: CGRP, adrenomedullin and adrenomedullin 2 display endogenous GPCR agonist bias in primary human cardiovascular cells
Source: Commun Biol. 2021 Jun 23;4:776. doi: 10.1038/s42003-021-02293-w (PMC8222276; doi:10.1038/s42003-021-02293-w)
Supplement: Supplementary file 3 — Description of Additional Supplementary Files [file 42003_2021_2293_MOESM3_ESM.pdf]

Description of Additional Supplementary Files

File name: Supplementary Data 1

Description: *Data for Figure 1 panels a-f – HUVEC*

Raw data for expression and signaling properties of wild type HUVECs following stimulation with CGRP, AM and AM2. This data enables the generation of panels a-f from Figure 1. Panels g, h, i are derived from the data in these files so are not included.

File name: Supplementary Data 2

Description: *Data for Figure 2 panels a-e – HUVEC inhibitors*

Raw data for signaling properties of HUVECs treated with various intracellular signaling inhibitors (PTX, Rp-8-Br-cAMP, YM-254890, ESI-09) and either cAMP or ERK1/2 responses to stimulation with CGRP, AM and AM2 assayed. This data enables the generation of panels a-e from Figure 1.

File name: Supplementary Data 3

Description: Raw genome sequencing results, as provided by Euorgentics for section of exon 1 of RAMP2 gene containing sgRNA target sites for Control HUVECs. Sequenced using the RAMP2 primer: GCCTATTTTCGGAGGGTCTC.

File name: Supplementary Data 4

Description: Raw genome sequencing results, as provided by Euorgentics for section of exon 1 of RAMP2 gene containing sgRNA target sites for CRISPR-Cas9 RAMP2 knockout. Sequenced using the RAMP2 primer: GCCTATTTTCGGAGGGTCTC. The data here demonstrates heterogeneity indicative of a mixed pool of cells with different genome edits to the RAMP2 open reading frame.

File name: Supplementary Data 5

Description: *Data for Figure 3 panels a-f – HUVEC RAMP1*

Raw data for expression and signaling properties of HUVEC expressing RAMP1 following stimulation with CGRP, AM and AM2. This data enables the generation of panels a-f from Figure 3. Panels g, h are derived from the data in these files so are not included.

File name: Supplementary Data 6

Description: *Data for Figure 4 panels a-e – HCM*

Raw data for expression and signaling properties of HCMs expressing RAMP1 following stimulation with CGRP, AM and AM2. This data enables the generation of panels a-f from Figure 4. Panels g, h are derived from the data in these files so are not included.

File name: Supplementary Data 7

Description: *Data for Figure 5 panels c – AM2 potencies*

Raw values for the Log potency of AM and CGRP at CGRPR and AM1R. Values have been obtained from various other studies and enable comparison of our cell line signaling properties.
