# Supplementary material for: CGRP, adrenomedullin and adrenomedullin 2 display endogenous GPCR agonist bias in primary human cardiovascular cells
Source: Commun Biol. 2021 Jun 23;4:776. doi: 10.1038/s42003-021-02293-w (PMC8222276; doi:10.1038/s42003-021-02293-w)
Supplement: Supplementary file 11 — Reporting Summary [file 42003_2021_2293_MOESM11_ESM.pdf]

nature research

Corresponding author(s): Graham Ladds

Last updated by author(s): Feb 26, 2021

Reporting Summary

Nature Research wishes to improve the reproducibility of the work that we publish. This form provides structure for consistency and transparency in reporting. For further information on Nature Research policies, see our Editorial Policies and the Editorial Policy Checklist.

Statistics

For all statistical analyses, confirm that the following items are present in the figure legend, table legend, main text, or Methods section.

n/a	Confirmed	
☐	☒	The exact sample size (*n*) for each experimental group/condition, given as a discrete number and unit of measurement
☐	☒	A statement on whether measurements were taken from distinct samples or whether the same sample was measured repeatedly
☐	☒	The statistical test(s) used AND whether they are one- or two-sided<br>*Only common tests should be described solely by name; describe more complex techniques in the Methods section.*
☒	☐	A description of all covariates tested
☐	☒	A description of any assumptions or corrections, such as tests of normality and adjustment for multiple comparisons
☐	☒	A full description of the statistical parameters including central tendency (e.g. means) or other basic estimates (e.g. regression coefficient) AND variation (e.g. standard deviation) or associated estimates of uncertainty (e.g. confidence intervals)
☐	☒	For null hypothesis testing, the test statistic (e.g. F, t, r) with confidence intervals, effect sizes, degrees of freedom and P value noted<br>*Give P values as exact values whenever suitable.*
☒	☐	For Bayesian analysis, information on the choice of priors and Markov chain Monte Carlo settings
☒	☐	For hierarchical and complex designs, identification of the appropriate level for tests and full reporting of outcomes
☒	☐	Estimates of effect sizes (e.g. Cohen's *d*, Pearson's *r*), indicating how they were calculated

Our web collection on statistics for biologists contains articles on many of the points above.

Software and code

Policy information about availability of computer code

Data collection	Data was collected using MikroWin 2000 Software (Mithras LB 940 platereader) and exported as Excel files (cAMP, NO, pERK1/2 and cell proliferation). Ca2+ data was measured using BD Pathway 855 Bioimaging Systems running Attovison Software and exported as Excel files. qPCr was performed using 7900HT Fast Real-Time PCR System and analysed using Relative Quantification software.
Data analysis	Data was analysed using Graphpad prism 8.4 for three parameter and operational model fitting of dose response data. Bias plots and error calculations were performed using Excel for Mac v16.48

For manuscripts utilizing custom algorithms or software that are central to the research but not yet described in published literature, software must be made available to editors and reviewers. We strongly encourage code deposition in a community repository (e.g. GitHub). See the Nature Research guidelines for submitting code & software for further information.

Data

Policy information about availability of data

All manuscripts must include a data availability statement. This statement should provide the following information, where applicable:

- Accession codes, unique identifiers, or web links for publicly available datasets
- A list of figures that have associated raw data
- A description of any restrictions on data availability

All raw data will be posted on the university of Cambridge repository server and made freely available upon request.

Field-specific reporting

Please select the one below that is the best fit for your research. If you are not sure, read the appropriate sections before making your selection.

- [x] Life sciences
- [ ] Behavioural & social sciences
- [ ] Ecological, evolutionary & environmental sciences

For a reference copy of the document with all sections, see nature.com/documents/nr-reporting-summary-flat.pdf

Life sciences study design

All studies must disclose on these points even when the disclosure is negative.

Sample size	All sample sizes were determined using the guidelines from the British Journal of Pharmacology (instructions to authors and the published guidelines editorial (Curtis M et al Br J Pharmacol. 2018 Apr;175(7):987-993).
Data exclusions	All data analysis adhered to the guidelines described in - Curtis M et al Br J Pharmacol. 2018 Apr;175(7):987-993. Data exclusion was not performed except for instances where control experiments failed and then the entire experiment was removed from analysis. E.g if Ionomycin failed to generate a Ca2+ response then the data for agonists at the CLR was not analyzed.
Replication	When using primary cell lines, there is a degree of variation in the precise potency and extend of signalling magnitude. All experiments were appropriately controlled using "system pathway agonists". Should these "pathway controls" generate in appropriate responses then the entire data set was removed. Tolerance for variation was considered to be <3-fold changes in potency for the system parameters tested.
Randomization	Randomization in this study was not performed due to the limited number of reagents and cell lines used. All data generated is entirely novel and signalling pathways not previously explored. As such there was no "preconception" of what "should have happened". To ensure robustness of data, independent members of the research team preformed some overlapping experiments to determine reproducibility. On occasion the experimentalist was not informed of the compounds used or cells lines investigated.
Blinding	See comments on randomization regarding some aspects of blinding.

Reporting for specific materials, systems and methods

We require information from authors about some types of materials, experimental systems and methods used in many studies. Here, indicate whether each material, system or method listed is relevant to your study. If you are not sure if a list item applies to your research, read the appropriate section before selecting a response.

Materials & experimental systems

n/a	Involved in the study	
☐	☒	Antibodies
☐	☒	Eukaryotic cell lines
☒	☐	Palaeontology and archaeology
☒	☐	Animals and other organisms
☒	☐	Human research participants
☒	☐	Clinical data
☒	☐	Dual use research of concern

Methods

n/a	Involved in the study	
☒	☐	ChIP-seq
☒	☐	Flow cytometry
☒	☐	MRI-based neuroimaging

Antibodies

Antibodies used	Detection of primary Cas9 protein - using Cell Signalling Technology Ab 7A9-3A3 (mouse)<br>Secondary antibody to the mouse Cas9 primary (Invitogen anti-Mouse IgG antibody, Alexa 488)
Validation	Cas9 antibody has been validated by Cell Signalling technology (https://www.cellsignal.co.uk/products/primary-antibodies/cas9-7a9-3a3-mouse-mab/14697). 43 current citations<br>Secondary antibody anti-Mouse IgG antibody, Alexa 488 validated by Invitrogen (https://www.thermofisher.com/antibody/product/Goat-anti-Mouse-IgG-H-L-Cross-Adsorbed-Secondary-Antibody-Polyclonal/A-11001)

Eukaryotic cell lines

Policy information about cell lines

Cell line source(s)	HUVECS, HUEACs and HCMs all from at least 3 individual donors were sourced from PromoCell
Authentication	HUVECs were validated as described by Promocell (https://www.promocell.com/f/product-information/manual/C-12250.pdf)<br>HUEACs were validated as described by Promocell (https://www.promocell.com/f/product-information/manual/C-12252.pdf)

	HCMs were validated as described by Promocell (https://www.promocell.com/f/product-information/manual/C-12811.pdf)
Mycoplasma contamination	All cell lines that enter the lab are firstly tested for microplasma contamination. All passed.
Commonly misidentified lines<br>(See ICLAC register)	None
